# Perception of the Impact of COVID-19 on a Sample of Spaniards with Hearing Disabilities

**DOI:** 10.3390/ijerph20021460

**Published:** 2023-01-13

**Authors:** Mª Ángeles Martínez Sánchez, Antonio Muñoz-García, Cristina Ros Gil

**Affiliations:** 1 Department of Social Work and Social Services, University of Granada, 18071 Granada, Spain; 2 Department of Developmental and Educational Psychology, University of Granada, 18071 Granada, Spain; 3 Faculty of Psychology, University of Granada, 18071 Granada, Spain

**Keywords:** hearing impairment, COVID-19, communication difficulties, deaf people’s perceptions

## Abstract

This paper describes an empirical study carried out with 40 Spanish deaf people, users of sign language, between 19 and 45 years of age, which gathers their perceptions of aspects related to the incidence of the COVID-19 pandemic and its repercussions. During the pandemic, people with hearing disabilities, among other groups, were forgotten. They suffered from accessibility problems to the information issued by the authorities, violating their right to be informed and exposing their health to COVID-19. In this work, we identify the problems they suffered and what effects COVID-19 had on their lives. This will help to take the appropriate measures to restore their rights and design policies and strategies to deal with any new future health emergency. For this, an ad hoc questionnaire was designed, adapted to easy reading and sign language. This was publicized via email and WhatsApp through the Association of Deaf People of Granada and Province (Spain) and was responded to online and by video call with the collaboration of sign language interpreters using the LimeSurvey platform. The results discover (1) the difficulties of communication barriers in the relationship with health professionals and institutions, as well as in the spheres of work and education, (2) similarities with the rest of the population in the negative effects of confinement, and (3) presence of positive effects, such as the development of positive activities and emotions. The study highlights the need to increase economic and institutional support aimed at improving coping resources, access to information, and the reduction of social and institutional barriers that would allow people with hearing disabilities to successfully face future health problems of a global nature such as that experienced with COVID-19.

## 1. Introduction

Since the COVID-19 outbreak was declared a global public health emergency by the World Health Organization (WHO) on 30 January 2020, the pandemic has had significant and long-term societal implications for the economy, education, and employment. Al-though the consequences arising from the state of alarm proclaimed in many countries, and from the disease itself, have had global repercussions, they have not affected everyone in the same way [1]. People in vulnerable situations, and especially those with disabilities, have been disproportionately affected due to social and institutional barriers that hinder adequate responses to situations caused by COVID-19 [2].

The Global Monitor on the Rights of Persons with Disabilities for COVID-19 [3] has highlighted the great injustices suffered by people with disabilities around the world during the first stage of the pandemic, including the strict measures adopted in residential resources, the interruption of essential services in the community, and the damage in different areas, as well as the denial of access to medical care [4]. In response to this failing in countries such as China, Iran, Latin America, the Caribbean, and Spain, studies have been carried out on the case of people with disabilities in the situation caused by COVID-19 [1], which highlighted the need for awareness of the risks that this health situation poses for these groups and analyzed the responses that have been implemented to reduce its impact.

Studies on deaf people have been aimed at identifying the barriers deaf people have faced, how they have been resolved, and recommendations for their governments [5]. Also of interest is the study carried out in Italy [6], centered on the context of hospitals [6], which highlights the need to focus attention on people with hearing loss who “experience an additional condition of isolation in addition to the international social distancing and the Italian quarantine” [6]. It also points out the difficulties for deaf people in understanding and communicating with health personnel who use personal protective equipment which covers their faces. It is true that “masks offer protection against the virus but prevent lip reading and reduce acoustic transmission” [6]. Attempts have been made to resolve this situation at the international level by proposing the use of masks with a transparent plastic window over the mouth and mobile voice and text applications [2].

The studies on deaf people have shown that the vulnerability of deaf people is precisely related to the limitation of access to information on disease control, regulations, access to health resources, and educational resources, among others [2]. In response to this situation, some countries have proposed a series of recommendations. The United States, through the Office of Civil Rights of the Department of Health and Human Services, published a bulletin in which it also provided “guidance to the authorities to ensure the dissemination and accessibility of information and communications to persons with disabilities, in pursuit of their equal opportunities to benefit from emergency response efforts” [2] (p. 2). In the Philippines, the “Human Rights Commission has published information to support health agencies in adapting public messages for vulnerable groups in communities, including children and people with disabilities” [2] (p. 2). In addition, the United Nations has recommended, among others, procedures “to ensure that information on measures related to COVID-19 is accessible to people with disabilities, by using sign language, subtitles and easy to read adaptation, among others” [2] (p. 4).

Governments, such as those of Spain or China, had a slow response to this need. In Spain, when the state of alarm was declared by the government, it was not adequately disseminated to the deaf community, with the consequences that this may have had on the spread of the virus and the damage to the health of both deaf people and the rest of society. The first response to this situation in both China and Spain was from civil society and grass-roots social organizations, who responded appropriately to the needs of the people [5,7]. A solution consisted of providing valid and usable informative materials for deaf people, such as a sign language translator, electronic formats, and videos in sign language disseminated through different platforms [8,9,10]. In Spain, social organizations for deaf people, such as the National Confederation of Deaf People of Spain, social organizations for families, such as the Spanish Confederation of Families of Deaf Peoples [11,12], and social organizations for people with cochlear implants, such as the Federation of Cochlear Implants Associations [13], demanded from the authorities that all messages be accessible to all deaf and deafblind people—with audiovisual materials in SL and with subtitles, as well as the incorporation of rigorous subtitles, in real-time, in all official appearances and information about COVID-19—and that 112/emergency calls and telephones numbers for reporting symptoms offer the possibility of using text messaging applications (e.g., WhatsApp) or written text.

Studies of deaf people in the United Kingdom [14,15] concluded that the life expectancy of deaf people (in developed countries) is 5 years less than that of hearing people due to inadequate access to health information. To overcome this problem, digital systems and human resources (SL interpreters) can be used. In the immediate future, the protection framework should be adapted to the specific needs of people with disabilities to improve policies aimed at alleviating the impact of the pandemic [16].

In the educational environment, most States have temporarily closed educational institutions, affecting children, teachers, and families, who have had to adapt in record time from a face-to-face teaching model to a teaching model at a distance, at times without resources to adapt to it, thus generating gaps in access, use, and schooling [17]. In this sense, the World Federation of Deaf People (WFD) made a statement [18] through which it stated that “during the COVID-19 pandemic, many schools have closed and/or moved online. This includes both deaf schools and local schools; however, during the pandemic local schools have especially struggled to meet the needs of deaf learners” (p. 1). This study also shows the lack of resources adapted to communication needs, demanding compliance with international standards, such as the UN Convention on the Rights of Persons with Disabilities or the Convention on the Rights of the Child, which urge governments to guarantee the access of deaf students to information and education in national sign languages. The document also states that “qualified teachers who are proficient in national sign languages are essential for the success of virtual learning” (p. 2). In addition, the WFD “calls on all governments to ensure deaf children and youth receive equitable access to information and education in national sign languages during and after the pandemic, including access to instruction by sign language-proficient teachers and the provision of visual learning materials” (p. 3). To this end, the WFD endorses the promotion of accessible interactive activities as an alternative to face-to-face contact, although it warns that this group is especially vulnerable to online abuse and exploitation. The same document affirms the need for students, families, and teachers to receive clear guidelines for safe use of social networks and the Internet.

In order to improve these policies, it is important to understand the way in which the confinement situation and its repercussions affected the lives of deaf people, with particular regard for the aspects related to issues of communication.

With this objective, we propose this descriptive and cross-sectional study, carried out on a local level with the collaboration of the Association of Deaf People of Granada and Province (Spain) and other organizations of the associative movement, aimed at (1) determining how the situation caused by COVID-19 has affected a group Spanish deaf people, be it students or workers, in the personal, social, and work spheres; and (2) identifying aspects that could account for the existence of a situation of vulnerability compared with the rest of the population, fundamentally due to the presence of communication barriers.

## 2. Materials and Methods

### 2.1. Participants

This research is carried out on a sample of signing deaf people, sign language users (who use sign language to communicate) [19], through an ad hoc questionnaire.

“Deaf people” are identified as those who often have profound hearing loss, meaning they hear little or nothing and most communicate using sign language [20]. In addition to linguistic information, deaf and hard-of-hearing people rely heavily on facial information, expressions, lip reading, and other visual input to support speech comprehension [21].

Deafness as a deficiency refers to the loss or abnormality of an anatomical and/or physiological function of the auditory system and has its immediate consequence in a hearing disability, which implies a deficit in access to oral language [22].

A total of 40 people (95% confidence level, 14.45% margin of error) participated in the study (37.5% men, 62.5% women), all deaf and of Spanish nationality, aged between 19 and 85 years old (m = 50.45 years, s = 16.84), with 25% older than or equal to 65 years of age, 32.5% between 50 and 64 years old, and 20% between 30 and 50 years old. The people participating in the study specified that they lived in the city of Granada (60%) or its province (40%), and 40% reported living in a rural context vs. urban.

Forty per cent of the participants were married and 25% single, 12.5% reported living with a partner, 10% divorced, 5% separated, and 7.5% widowed.

Of the participants, 27.5% indicated that they had a recognized degree of disability in the range of 33–64%, and 72.5% stated that their degree of disability was equal to or greater than 65%.

Regarding educational level, 20% reported having less than 5 years of schooling, 25% completed primary or secondary education, and 20% higher education. Of the participants, 35% had a professional qualification or had undertaken vocational training (see Table 1, which shows the distribution of the sample according to their educational level).

With regard to occupation (see Table 2), 37.5% stated that they were working, and the same percentage identified themselves as retired or pensioner. In addition, 12.5% indicated that they were unemployed or subject to a Temporary Employment Regulation File, and only 10% identified themselves as a student.

Self-perception of their economic situation was considered as good or very good by 67.5% of the participants and bad or very bad by 32.5%.

In addition, 75% of the study participants reported living with at least one deaf person on a regular basis, 65% with one or two, and 10% with three or more. Of the group of participants, only one indicated that they had offspring with hearing disabilities who lived together at home.

### 2.2. Instruments

All the scales of the questionnaire were elaborated ad hoc. To choose the type of questions and their theme, firstly, a review of scientific studies was conducted, both theoretically and empirically oriented, on the subject of COVID-19 and its repercussions on the conditions and quality of life of people with disabilities, using academic databases held in the Web of Science. The quality of life, according to the Handbook on quality of life for human service practitioners [23], was also considered, as well as the Spanish manual of the GENCAT scale of quality of life [24].

A draft version of the questionnaire was reviewed by a person with hearing dis- abilities (SL user), paying attention to the comprehension of each of the questions in the questionnaire and making all the necessary modifications until an adequate comprehension was achieved. Terms whose meaning might be difficult to understand were adapted to easy language by including a box with the definition of the concept. For this, the team of professionals from the Down Syndrome Association of Granada was used; in addition, videos were included for each item where the text of the question was adapted to SL by a professional Sign Language Interpreter (SLI). A final version of the questionnaire was reviewed by four adults with AD (auditory disability) and consisted of the questions described below. The three authors of this article also acted as experts throughout the process at different times, assessing the representativeness, clarity, and relevance of each of the items, the scoring system, and the measurement scale used and modifying the necessary aspects until a consensus was reached.

Socio-demographic aspects. For the characterization and understanding of the socio-demographic aspects of the participants with AD, five questions were defined about sex (man, woman, or other), age, nationality, marital status (married, single, widowed, separated, divorced, or other), occupation (working, retired or pensioner (has worked before), retired or pensioner (has not worked before), unemployed but has worked before, unemployed and looking for first job, unpaid domestic work, student, work-study, or other).

Participants were also asked to indicate the degree of disability by selecting one of four possible options (i.e., less than 33%, in the range of 33–64%, 65% or more, or other), as well as the number of people with hearing disabilities with whom they live (with 5 possible options: none, one, two, three, or other), and place of residence.

One question designed by the Sociological Research Centre [25] was also included in order to establish educational level, and two additional questions adapted from a study conducted on people with intellectual and developmental disabilities [1] collected information on the home environment (own house, family home, shared house or flat, residence, or other) and the economic situation (options: very bad, bad, or good).

Living conditions during the pandemic and impact of COVID-19. Five questions were adapted from the study promoted by the institution Full Inclusion on COVID-19 and Intellectual and Developmental Disabilities [1]: the place of residence during confinement (own house, family home, shared house or flat, residence, or other), having been sick with COVID-19, performing a COVID-19 test (no; yes, but it had a negative result; or yes, with a positive result although it later turned out to be negative), having been in isolation (yes, no, or no response), and having been in the hospital or health centre for the coronavirus.

Two additional questions were elaborated to ascertain the place where the confinement was experienced during the pandemic (in one’s own home, the home of relatives, friends, residential centre, or other), and the characteristics of the family nucleus of residence during the confinement (single-parent (mother or father with children), two parents with one child, two parents with two children, large family, or people from the extended family).

A question on a six-point Likert scale, ranging from 1 to 5 (1 = very bad, 5 = totally well), was used for scoring the emotional feeling during the isolation situation. In addition, a five-point Likert-type scale (1 = very bad, 5 = very good) assessed the treatment received in the hospital or health centre.

Finally, 12 items with a 5-point Likert-type response format (1 = Nothing, 5 = Much), grouped for this sample in a single factor, explained 69.79% of the variance with a Cronbach’s Alpha of 0.96, assessing the degree to which each of the proposed measures would improve, in the respondent’s opinion, the quality of life of people with hearing impairment:

- For emergency telephone numbers (061, 112…) to be directly accessible in writing or by video call.
- For communication between the patient and the health system to be facilitated through instant messaging or written text.
- For health emergency telephone numbers and others to communicate that you have symptoms of COVID-19, carry out follow-ups, etc., to offer the possibility of using instant messaging or written text.
- Improve access to health information. Guarantee an adequate state of health, compliance with guidelines, and/or treatments.
- Monitoring of health status so that health is not threatened due to lack of information.
- Generalization of the use of approved transparent masks by those who serve the public in order to guarantee accessibility for deaf people. Opaque masks do not allow one to read lips or interpret emotions.
- For the audiovisual communication media to rigorously broadcast information with subtitles and in SL in a window where the interpreter is of adequate size and resolution.
- Facilitate complete accessibility to online videos, where there is clarity and synchronization of text and voice to facilitate total understanding of the message.
- For official live communications from the authorities (health, politics, etc.) to be accessible in SL and with live voice-to-text subtitles.
- Improve the means for people with hearing disabilities to access remote (online) education: SL interpreter, subtitled classes, visualization of the teachers’ lips, sufficient and adequate explanations for students with deafness, sufficient print media materials, and videos with rigorous subtitling.
- Improve the conditions for working from home: with SL interpreters, online meeting transcription, and government-subsidized speech-to-text facilities so that access to working life is not impeded.
- Install audio induction loop systems to enable communication and access to information for those who use hearing aids and cochlear implants.
- Financially guarantee the maintenance of hearing aids (batteries, spare parts, etc.).

Aspects of communication. Five questions were designed regarding various aspects related to health institution professionals in order to establish the personal evaluation of communication with them (impossible, very difficult, difficult, fair, acceptable, good, very good, or totally satisfactory), whether an SLI was needed (never, rarely, often, always, or no response), the availability of an SLI when it was needed (whenever I have needed it, when the health centre or hospital has arranged it, no, or no response), the possibility of communicating with the family or having an SLI when it was needed in the case of having been hospitalized (whenever it was needed, when it was arranged in the hospital, no, or no response), and the medium in which information was received during the pandemic (in SL, oral, written, or other).

Three questions from the COVID-19 and intellectual and developmental disabilities study [1] were also adapted regarding the sources of information used (organizations/associations, media, social networks and the internet, friends, work/school centers, or other), receipt of information on coronavirus (yes or no), and degree of comprehension of the information received on a 10-point scale (1–10).

Evaluation of the effects of the situation of confinement or the pandemic on living conditions. Firstly, participants were asked about the effect of the pandemic on the performance of 21 proposed activities, indicating in each case one of three proposed answers (i.e., I used to do it before and now I don’t do it anymore, I used to do it before and now I continue to do it, or I have started doing it during this time).

Secondly, the participants were asked to rate on a 5-point Likert scale (1 = Not at all, 5 = A lot), the degree to which 30 proposed situations had worried them during the time of confinement: concern about the coronavirus and its consequences for one’s own or someone else’s health, situations associated with one’s own or someone else’s illness and possible hospitalization (for example, the illness of a family member or person with whom I live, the consequences of hospitalization), low mood (e.g., feeling little interest in doing things), feeling restless (e.g., feeling nervous), fear (e.g., fear of a serious illness), and loneliness (e.g., being alone).

Participants were also asked to report whether they had experienced (yes or no) each of the following 10 proposed feelings: safety, tranquility, relief, responsibility, solidarity, fear, uncertainty, anguish, impotence, and concern.

In addition, they were required to indicate what effects, among 5 proposed (i.e., I continue with normal life, changes in sleep, mood swings, changes in routine, thinking about participating in solidarity actions, or others) the pandemic situation has had on their lives.

Six possible measures (i.e., receive more information, more or less strict measures by the Government, prohibit and punish the spread of misleading and unfounded information, or receive social and/or economic support from associations and institutions) were also proposed for the respondents to assess whether they (yes or no) would reduce the effect of a future pandemic on the lives of deaf people.

Questions 10a and 10b were adapted from the Sociological Research Centre [26,27] regarding the degree to which everything that happened with the pandemic has affected the participant, in particular the effects on personal and social life, as was Question 12, relating to the measures taken in an additional personal capacity to those recommended by the health authorities.

Based on the study, COVID-19 and Intellectual and Developmental Disabilities [1], and targeting students in particular, four questions were developed specifically aimed at establishing the influence of the pandemic on students: (1) whether they experienced difficulty in six proposed aspects (i.e., the understanding of explanations or tasks, insufficient time to complete them, attention/concentration problems, interaction with the media, learning support, or others), (2) whether they adapted to the distance learning situation (yes, no, with some difficulty, or it has not been necessary), (3) whether they received any help to receive distance training (yes or no) and, (4) if so, who provided it (teachers, a family member, or other).

In order to establish the influence on workers with AD, taking as reference previous studies on the effect of the pandemic on the living conditions of people with disabilities as well as institutional internet sites with information on COVID-19 and people with AD (e.g., CNSE, FIAPAS, ASOGRA, and ASPRODES), participants were asked two questions: (1) whether it had been possible to work during the confinement (yes—in person, yes—remotely or online, no, or other), and (2) how COVID-19 has affected employment (it has not affected me, I am temporarily laid off, I have lost my job and I doubt I will be able to return to work soon, it has given me a job opportunity and I have started working, or other).

### 2.3. Questionnaire Response Procedure

The participants received an explanatory email message sent by the Association of Deaf People of Granada and Province, informing them of the characteristics of the study and its objectives. This provided a link to an online assessment protocol using the LimeSurvey platform.

Other people were called by telephone or by video call by the association, responding in person and individually and in SL to the questionnaire adapted to SL, their responses being recorded on the platform by the researcher. The study was carried out in accordance with the ethical guidelines indicated by the Declaration of Helsinki and the Spanish Act on the Protection of Personal Data. It also complied with the ethical stan-dards of the University of Granada.

## 3. Results

The results of the research will allow us to determine how COVID-19 has affected the quality of life of the group of deaf people.

Of the participants, 17.5% reported having been infected by coronavirus diagnosed by carrying out a test, compared with 12.5% who indicated that they did not know whether they might have been infected. In addition, 32% of participants indicated that they had not taken any tests.

Ten per cent of the people who participated in the study had to go through a situation of isolation. When asked about the way they felt during that period of confinement, apart from the 15% who indicated that they had not experienced any emotional change, 20% felt bad or very bad, compared with 7.5% who indicated that they felt quite well.

Sixty-five percent of the sample was not in hospital or in their health centre due to COVID-19. The mean evaluation rating of the treatment received in hospital by those who used it was 2.87 (range 1–5, s = 1.45). Although 15% of users indicated the lowest score (i.e., 1), 35% evaluated the care received with a score of 3 or more, and 20% with 4 or 5.

**Figure 1:**
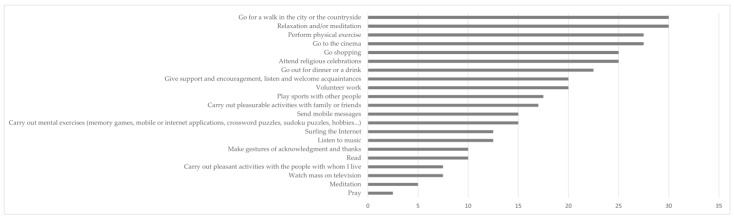
Percentage of people who have stopped doing the indicated activities.

The impact of the pandemic situation on lifestyle was carried out by assessing the way in which the participants perceived changes in the degree of performance of certain activities, which might be abandoned as a result of the pandemic, might continue to be carried out, or might even be a new activity for the person. The impact of the pandemic on the execution of the proposed activities (see Figure 1) indicated the abandonment of those activities of a social nature generally carried out outside the home, with the exception of relaxation and/or meditation. On the other hand, the pandemic situation had very little impact on individual activities that could be carried out in one’s own home and that were already being carried out.

**Figure 2:**
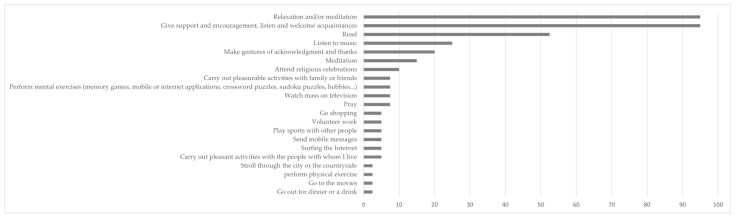
Percentage of people who have started doing the task as a result of the pandemic.

The change in living conditions imposed by the pandemic also affected the types of activities carried out (Figure 2), which shows, on the one hand, how the pandemic led to an increase in activities directly related to improving physical, psychological, and spiritual well-being.

**Figure 3:**
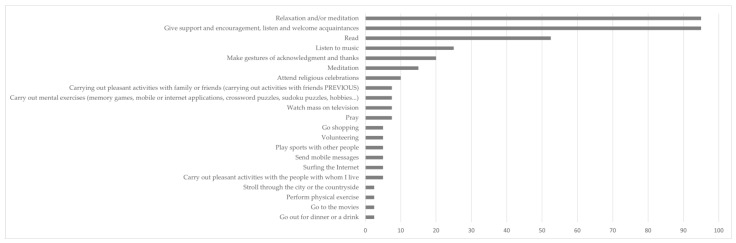
Percentage of people who carried out the activity before the pandemic and continue to do so.

However, the easing of isolation measures and the progression towards a more normalized situation has meant resuming the performance of social activities carried out outside the family sphere and a reduction of activities of an individual nature that were being carried out during the confinement (see Figure 3). It should be noted that among the individual activities that have been least affected by these changes are the majority of those that provide an intrinsic cognitive or spiritual benefit (e.g., performing pleasurable activities with family or friends, praying, performing mental exercises, watching Mass on television, etc.).

The evaluation of the degree of concern that particular aspects associated with the pandemic situation have caused indicated that factors related to the fear of contagion and its consequences, followed by other more specific aspects associated with the effects on health and physical well-being, were those that aroused greater concern (see Table 3). Next were indicated the concerns related to aspects of psychological well-being, with the fact of being or feeling alone as the least worrying situations. Seventy-five percent of the specified situations involved an average concern higher than the possible average value of 2.5 points, without much variability being observed among the responses of the participants.

**Figure 4:**
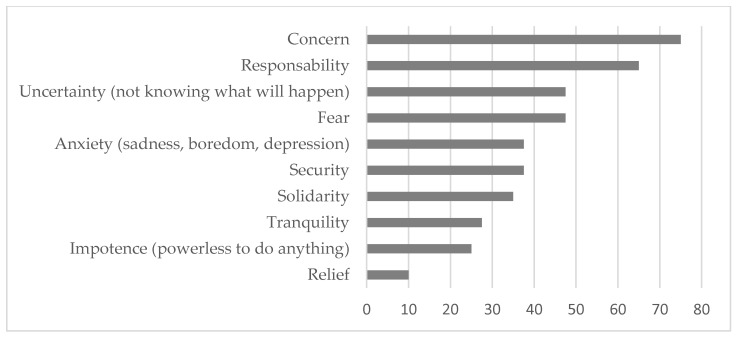
Percentage of people with AD in whom the pandemic situation has generated positive and negative feelings.

The pandemic situation also evoked certain feelings in the participants, associated mostly with negative emotions (see Figure 4). In addition to arousing negative feelings of concern, uncertainty, or fear, living through the situation also aroused, although to a lesser extent, feelings of responsibility. The lived experience also provided positive feelings in a limited way (less than 50% of people with AD), expressed in the form of security, solidarity, tranquility, or relief. There were also very few who indicated having felt powerless in the face of this situation.

**Figure 5:**
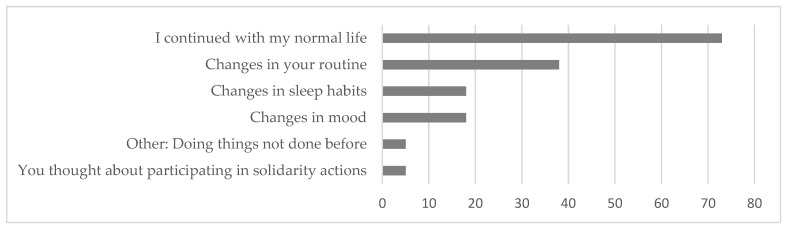
Impact of the pandemic on the daily life of people with AD.

The pandemic situation was perceived by the study participants as not having a great impact, since 72% reported having continued with their normal lives (see Figure 5). However, it has had an impact to a certain extent by altering the daily routine or causing changes in mood or sleep habits. To a lesser degree, the situation prompted feelings of aid towards others or influenced the start of new behaviors in this group of deaf people.

**Figure 6:**
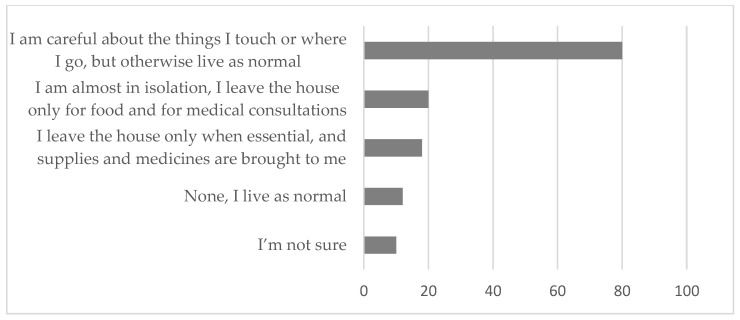
Adoption of measures in a personal capacity to control the pandemic.

When asked about the measures taken to control COVID-19 beyond the official measures suggested by the authorities, 80% of the participants reported being careful with the things they touch or where they go (see Figure 6). Less than 20% of the participants indicated having taken very restrictive measures, such as being almost in isolation, leaving the house only in cases of strict necessity, or even asking for help so as not to even have to go out shopping or to the doctor. All shared the idea that this situation broke the normality of daily life.

The pandemic has generally affected most of the participants, this influence being valued as “quite a lot” or “a lot” by 70% of them. Twenty percent indicated “a little”, and only 10% indicated that they had not been affected at all by everything that the pandemic entailed.

**Figure 7:**
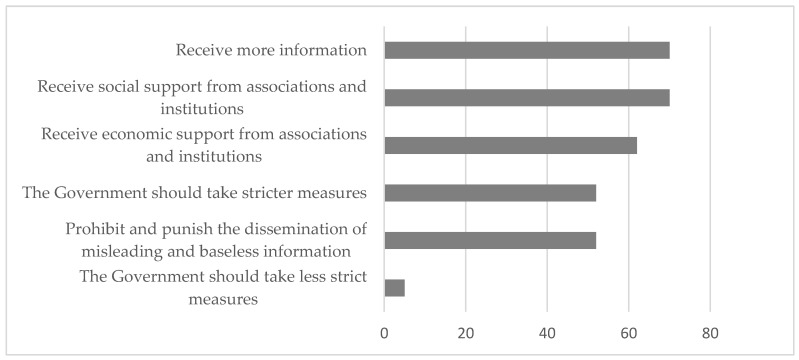
Percentage of agreement of the participants with measures to be taken in the case of a new pandemic.

In relation to the measures that it would be necessary to adopt in the case of a future pandemic, which were aimed at reducing its impact on the lives of people with hearing disabilities, 70% of those surveyed indicated the need to receive greater social support from associations and institutions, as well as more information (see Figure 7). In addition, 62.5% suggested receiving financial support, and 52.5% of the participants indicated the need to prohibit and punish the dissemination of misleading and baseless information, as well as the need for government to take stricter measures. Only 5% thought that the government should reduce the limitations imposed to control the pandemic situation.

In addition to these measures, the study participants all suggested the need to improve accessibility by the use of transparent masks as well as always translating the information to sign language, especially in health institutions and in all matters concerning the relationship with health professionals, also suggesting increasing the number of interpreters and the quality of interpretation.

The impact of the pandemic situation on social life has been moderate, with more than a third of participants (38.5%) considering that the pandemic situation has somewhat affected their social lives, with 42.5% assessing this impact as quite a lot or a lot. Only 12.5% indicated an absence of impact on social life, and 7.5% were unable to assess this impact.

The pandemic situation has affected the personal lives of one-quarter of the people participating in the study, with 25% reporting not being able to assess this impact or not having been affected. On the other hand, 42% indicated that their personal life had been affected a lot or quite a lot, and 32% indicated that the pandemic had affected their personal life to some extent.

In relation to aspects concerning communication associated with this pandemic situation, among those participants who are regular users of SL, 72.5% required the support of an SLI often or always, compared with 2.5% who never needed an interpreter. Twenty percent indicated having needed one a few times. If we take into consideration the assessment of communication with the health centre or hospital workers, this represented an average value of 2.57 (range 1–5, s = 1.37). In addition, the degree of comprehension of the information received was 5.93 (s = 2.66) on a 10-point scale (1–10), with 37.5% of responses below the value of 3, and only 20% of subjects indicating scores equal to or greater than 4.

The type of information received during the time of the pandemic was largely through SL (Table 4), although diversity was observed in the different media used as an augmentative resource of communication.

**Figure 8:**
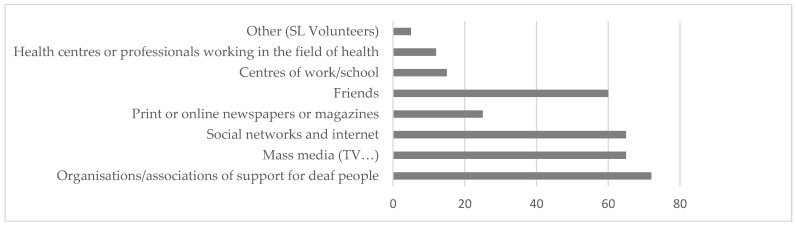
Percentage of use of information sources.

Regarding the sources of information (see Figure 8), the most used were the associations/organizations that support deaf people, the media as well as social networks and internet and friends and, to a lesser extent, newspapers or magazines, the workplace, and health centers or health professionals or SL volunteers.

Although 95% reported having received information about the coronavirus (e.g., what it is, how to prevent the disease, and how to look after our well-being), the degree of understanding of the information received during the pandemic concerning the coronavirus (e.g., how to reduce contagion and how to maintain good physical and mental health) was assessed, producing a mean score of 5.93 out of a maximum of 10 points (range 1–10, s = 2.66).

The evaluation of the importance of possible measures aimed at improving the quality of life of people with AD indicated the relevance of all the proposals, given that none of them obtained a mean score of less than 3 points out of a maximum of 5 (see Table 5). The aspects with the highest scoring were for the improvement of alternative communication resources for people with AD (i.e., subtitles, SL, and interpreters in windows of an adequate size) in order to improve audiovisual communication and distance education. Next in importance were all the measures regarding the improvement of procedures for communication related to health processes and institutions (e.g., medical visits, access to emergency rooms, and the option of exchanging text messages with institutions) in combination with technical improvements (synchrony of text and voice in online videos and use of transparent masks by medical personnel). Finally, the improvement of specific working conditions for people with hearing disabilities, financial support for the maintenance of hearing aids, and the installation of audio induction loop systems were evaluated.

The students who participated in the study indicated attention/concentration problems as the main difficulty in distance learning (50%). Interaction problems with the media (16.7%) and lack of support with learning (16.7%) were also mentioned. One person marked all the proposed answers as difficulties: difficulties in understanding explanations and tasks, insufficient time to complete the tasks, problems with attention/concentration, interaction problems with the media, and lack of support for learning.

Furthermore, 50% of the students indicated that they had not adapted to the distance training situation, and 71.4% had not received any type of help to receive this type of training. In the cases where help was received, it came from the teaching staff, although 33% stressed that they had not received help from anyone.

Of the workers participating in the study, 52.5% did not work at all during the confinement, either because they were retired (30%) or because they were unable to do so (22.5%). Of the remaining workers with hearing disabilities, only 12.5% did so in person, compared with 17.5% who worked remotely.

The impact of the pandemic situation on employment has been varied, although it had no effect on 27.5% of workers. Of the rest, 25% indicated that they were under some employment regulation system at the time of answering the questionnaire, whether it was a Temporary Employment Regulation File (22.5%) or an Adjustment of Employment Levels (2.5%). In addition, 7.5% had been affected by a Temporary Employment Regulation File, although their situation had returned to normal at the time of answering the questionnaire, 5% said they were retired, and 2.5% were unemployed.

## 4. Conclusions

The objective of this study was to establish the extent and impact of the change produced in the living conditions of deaf people due to the COVID-19 pandemic and, in particular, to the situation of confinement regarding aspects related to communication, distance education, work, and the repercussions on daily life.

Although in terms of health, the incidence of the disease can be considered similar to that of the rest of the population, the lived experience and the repercussions of the situation of confinement connected with the associated measures of protection have had significant consequences on their interaction with health services and professionals, and the information received by the media has been affected by communication difficulties and barriers.

In relation to aspects concerning communication associated with the incidence, perception, and experience of the pandemic situation for people with AD, our study completed the aspects addressed by another investigation [5] focusing on the study of quality of life. The results indicated the need, in order to reduce the impact of the crisis caused by the pandemic, for the affected deaf people to receive greater financial support from the State, the latter assuming the cost of maintaining hearing aids “since the group of people with disabilities is affected by notably higher unemployment rates, 25.2% compared with 15.1% in the community without disabilities” [28]. The results of this study are consistent with those of previous studies [2,5] in highlighting the necessity to improve resources for access to information, audiovisual communication, and distance education, in line with the manifesto of the World Federation of the Deaf [18]. This study also focusses on the need to test the veracity of the information available, prohibiting and punishing misleading information, in line with previous studies that indicate the disabled community`s greater vulnerability to misinformation [29].

Another significant aspect is the need to improve communication with health professionals by increasing the number of professional sign language interpreters, given that 72% of the people surveyed used sign language as a communication system. This would increase comprehension of the information available. Other aspects concerning communication—such as the use of subtitles and sign language interpreters to improve audiovisual communication and distance communication, the use of alternative systems to communicate and manage health care matters with medical institutions (request medical information, medical appointments, health control for COVID patients, emergency calls, etc.) through text messages, videos with voice and text synchronization, the use of induction loop systems as well as the use of transparent masks by the health personnel—would improve life at a personal level and reduce the negative impact on individuals’ lives of similar pandemic situations.

With respect to sources of information, the official media and the mass media should comply, in this particular case of deaf people residing in Spain, with current national legislation and international agreements, such as Law 33/2011 of 4 October, and General Public Health Law 27/2007 of 23 October, which recognizes Spanish sign languages and regulates the means of support for oral communication of deaf people, people with hearing disabilities, and the deafblind, or the United Nations Convention on the Rights of Persons with Disabilities, ratified by Spain and 162 other countries in order to protect the right to information and communication. In this connection, the results of the study could also be of great interest for the design of emergency plans and long-term policies aimed at adequately serving these groups in similar hypothetical situations in the future.

Regarding the impact of the pandemic situation on lifestyle, the way in which it has affected the performance of activities by deaf people has been similar to that of the rest of the population, above all affecting the performance of activities of a social nature undertaken outside the home [30,31,32]. As in the rest of the population, the use of technological resources for communication or carrying out activities at home (e.g., browsing the internet, playing games, listening to music, etc.) increased with the confinement situation [25]. As in another study [28], we found that activities related to the improvement of physical, psychological, and spiritual well-being were carried out to cope with the quarantine, although our study showed that it encouraged deaf people to start performing activities, such as relaxation and/or meditation, reading, or giving support and encouragement to other people, listening to them and hosting them in their home. Most of those who already carried out these activities did not stop doing them.

Concerning the impact on emotional aspects, the results were similar to that of previous studies [29] and CIS, where it was observed that approximately half of the study participants reported being affected emotionally and in their daily life due to the pandemic situation, although practically all indicated an increase in negative emotions. Our study bears out these results by verifying that there are many situations that are the subject of concern and to a similar degree, although the fear of contagion and its consequences was highlighted, followed by the effects on health, physical well-being, and psychological health. In the context of the feelings evoked by this situation, although the presence of negative feelings has been similar to that of other studies [27], reflecting a deterioration in mood [28], our study indicated, on the other hand, the increase in positive feelings of responsibility and, to a lesser extent, of solidarity and tranquility.

Regarding the situation of deaf students, in contrast to other studies where the distance learning situation did not seem to have had a great impact on the student [26], the participants in this study indicated difficulties of a technological nature and called for further development of educational support and guidance actions.

In relation to the limitations of this study, one of the important limitations has been the extension and comprehension of the questionnaire. On the one hand, the adaptation to easy reading has meant the introduction of definitions of concepts that are difficult to understand and, on the other hand, its adaptation to sign language, which has meant the introduction of videos, which has lengthened the completion time of the questionnaires. However, since we did not achieve a good online participation, we had to improvise the data retrieval process by allowing study participants to complete the questionnaire at the headquarters of the Association for Deaf People with the support of a sign language interpreter. We also suffered the problem of the use of masks that makes communication difficult, and it was a communication barrier. Likewise, we could not use transparent masks because they were not approved, and their effectiveness against the virus was not ensured. Assuming this new inconvenience, and following the recommendations of the group of experts, an agreement was reached to send the questionnaire by WhatsApp and via video call, with the collaboration of sign language interpreters. Although the sample size could be considered small, it has been proportionally larger than that of other national studies.

With regard to the orientation of the research, this has been similar to that of other studies [5], though expanded with the inclusion of additional aspects related to quality of life. Similarly, in the collection of data, interaction was facilitated through the use of technological support resources through text messaging and email. On analyzing the results obtained, we believe that it is justified to extend these implications to the rest of the autonomous communities of the national territory, identifying good practices, and providing arguments to the Spanish Government to establish a care plan for deaf people that identifies weaknesses and rules to follow in any future emergent pandemic situations that may occur. We also believe that the study should be extended to the European level to find common problems, weaknesses, or good practices and to ensure that the European Union legislates in a coordinated manner in terms of assistance to the deaf population and promotes common policies of action between the member countries in addition to an allocation of funds for achieving an inclusive European social space for all citizens. Despite all the difficulties described, this pioneering study has been carried out on a group of deaf people who use sign language to communicate. All these learnings arising from the systematization of the research methodology should be taken into account in future research carried out on the deaf population.

## Figures and Tables

**Table 1:** Distribution of the sample according to education level.

Education Level	*n*	Percentage	Accumulated Percentage
Less than 5 years of schooling	8	20.0	20.0
Primary education	6	15.0	35.0
Professional qualification	3	7.5	42.5
Compulsory secondary education	1	2.5	45.0
Intermediate level VET	3	7.5	52.5
Baccalaureate	1	2.5	55.0
Higher level VET	8	20.0	75.0
Diploma	5	12.5	87.5
University degree	3	7.5	95.0
Bachelor’s degree	1	2.5	97.5
Master’s degree	1	2.5	100.0

**Table 2:** Distribution of the sample according to their occupation.

Occupation	*n*	Accumulated Percentage
Working	15	37.50 (37.5)
Retired or pensioner (I have worked before)	11	27.50 (65)
Retired or pensioner (I have not worked before)	4	10.00 (75)
Unemployed but I have worked before	2	5.00 (80)
Unemployed and looking for my first job	1	2.50 (82.5)
Unpaid housework	1	2.50 (85)
Student	4	10.00 (95)
Record of Temporary Employment Regulation	2	5.00 (100)
Total	40	100.00 (100)

**Table 3:** Average concern generated for the participants in the study of different aspects associated with the pandemic situation.

	M ^a^	s
Consequences arising from the hospitalization of a family member	3.70	1.51
Fear of getting infected by the coronavirus	3.70	1.42
Fear that a family member or close acquaintance will be infected with the coronavirus	3.70	1.52
That there will be another pandemic	3.70	1.51
The death of a friend or acquaintance	3.65	1.53
Worry about the coronavirus and its consequences on my health	3.65	1.59
Receive medical care if necessary	3.63	1.58
The death of a family member or person I live with	3.60	1.72
Worry about the coronavirus and its consequences on the health of other people	3.57	1.55
Fear of having a serious illness	3.52	1.54
The consequences of hospitalization	3.47	1.59
The illness of a family member or person I live with	3.38	1.69
The alteration of my family relationships	2.88	1.64
The change in my daily life	2.85	1.66
Dying	2.85	1.79
The process of dying	2.83	1.88
Feeling nervous	2.75	1.72
Being unable to control my worries	2.73	1.71
Feeling down or hopeless	2.70	1.73
That life will continue as it is now	2.70	1.68
The alteration of my relationships with friends	2.65	1.49
Feeling little interest in doing things	2.63	1.60
Having nightmares	2.58	1.58
Changes in the way of being of my relatives or people I live with	2.47	1.58
Loss of my job	2.40	1.85
Feeling overwhelmed	2.28	1.65
Crying	2.25	1.61
Changes in my way of being	2.25	1.55
Feeling alone	2.18	1.60
Being alone	2.13	1.52

^a^ The range of scores is between 1 and 5.

**Table 4:** Type of information received during the time of the pandemic.

	*n*	Accumulated Percentage
In sign language	16	40.0 (40.0)
Oral	2	5.0 (45.0)
Written	5	12.5 (57.5)
In sign language, oral, and written	2	5.0 (62.5)
Sign language and subtitles	1	2.5 (65.0)
Sign language, written, and subtitles	1	2.5 (67.5)
Sign and written language	13	32.5 (100.0)

**Table 5:** Mean and standard deviation assessment of the importance of measures proposed to improve the quality of life of people with AD.

Measures Proposed to Improve the Quality of Life	Mean	SD
Audiovisual media with information with subtitles and in sign language in a window where the interpreter is large enough to be seen properly	4.10	1.87
More mechanisms for people with hearing disabilities to have access to a remote education (online)	4.10	1.68
More direct and faster access to health information that ensures an adequate state of health and follow-up	3.93	1.72
Emergency telephone numbers (061, 112…) for COVID-19, accessible by instant messaging or video call	3.80	1.51
Facilitate full accessibility to online videos with accuracy, articulacy, and synchrony of text and voice to enable complete understanding of the message.	3.80	1.72
Communication between the patient and the health system through instant messaging or written text	3.73	1.42
Online videos with synchrony of text and voice that enable the complete understanding of the message	3.70	1.40
That the health emergency telephone numbers and those supplying information on COVID-19 give the option of using instant messaging or written text	3.68	1.62
Use, by those serving the public, of transparent approved masks that facilitate lip-reading and interpretation of emotions	3.55	1.40
Conditions for working from home subsidized by the government. Sign language interpreters, transcription of online meetings, and voice-to-text facilities so that access to working life is not impeded.	3.45	1.37
Financially guarantee the maintenance of hearing aids (batteries, spare parts, etc.)	3.33	1.28
Installation of audio induction loops to enable communication and access to information for those who use hearing aids and cochlear implants.	3.18	1.24
